# Contribution of soil esterase to biodegradation of aliphatic polyester agricultural mulch film in cultivated soils

**DOI:** 10.1186/s13568-014-0088-x

**Published:** 2015-02-18

**Authors:** Kimiko Yamamoto-Tamura, Syuntaro Hiradate, Takashi Watanabe, Motoo Koitabashi, Yuka Sameshima-Yamashita, Tohru Yarimizu, Hiroko Kitamoto

**Affiliations:** National Institute for Agro-Environmental Sciences, 3-1-3 Kannondai, Tsukuba, Ibaraki 305-8604 Japan

**Keywords:** Aliphatic polyester, Biodegradable plastics, Esterase, PBSA

## Abstract

The relationship between degradation speed of soil-buried biodegradable polyester film in a farmland and the characteristics of the predominant polyester-degrading soil microorganisms and enzymes were investigated to determine the BP-degrading ability of cultivated soils through characterization of the basal microbial activities and their transition in soils during BP film degradation. Degradation of poly(butylene succinate-*co*-adipate) (PBSA) film was evaluated in soil samples from different cultivated fields in Japan for 4 weeks. Both the degradation speed of the PBSA film and the esterase activity were found to be correlated with the ratio of colonies that produced clear zone on fungal minimum medium-agarose plate with emulsified PBSA to the total number colonies counted. Time-dependent change in viable counts of the PBSA-degrading fungi and esterase activities were monitored in soils where buried films showed the most and the least degree of degradation. During the degradation of PBSA film, the viable counts of the PBSA-degrading fungi and the esterase activities in soils, which adhered to the PBSA film, increased with time. The soil, where the film was degraded the fastest, recorded large PBSA-degrading fungal population and showed high esterase activity compared with the other soil samples throughout the incubation period. Meanwhile, esterase activity and viable counts of PBSA-degrading fungi were found to be stable in soils without PBSA film. These results suggest that the higher the distribution ratio of native PBSA-degrading fungi in the soil, the faster the film degradation is. This could be due to the rapid accumulation of secreted esterases in these soils.

## Introduction

Plastics have spread and persisted around the world today because they have been widely used as basic materials in various industries. Used non-degradable plastic products cause waste management problems. Agricultural mulch films made have greatly contributed to the increase in the production of high-quality vegetables as they have been used to cover cultivated fields to maintain stable soil temperature and humidity, as well as to prevent weed growth. However, after harvesting, the recovery and recycling of used non-degradable mulch films would require a lot of energy and are labor intensive (Kyrikou and Briassoulis 2007).

Biodegradable plastics (BPs) have been developed as a possible solution to such environmental problems caused by the persistent plastic wastes. To date, various aliphatic polyesters that can be degraded by microorganisms in the natural environment have been commercialized as BP materials. Mulch films made from BPs are already in the market and are used to help farmers save time and labor as well as to reduce plastic wastes (Ngouajio et al. 2008). Chemical, physical, and biological degradability of BP mulch films are largely affected by the composition of the BP materials (Kyrikou and Briassoulis 2007). Although degradation speed of BP mulch films depends largely on environmental conditions (Kariyazono et al. 2000; Hoshino et al. 2001), the key factor controlling the degradation speed of BP mulch film in soil has not yet been elucidated. Knowledge of the mechanisms involved would enable the development of an indicator that would help farmers predict the degradability of mulch films under a given farmland condition before planting, and thus, select the suitable mulch film for their farm use.

Degradation of poly(butylene succinate-*co*-adipate) (PBSA) films (Bionolle® #3001) in three types of uncultivated soils (soil from extinct volcano crater, waste coal, and forest) had already been investigated (Nowak et al. 2011). To date, however, there are only a few papers reporting on the quantitative information about the degradation speeds of BP films and population size of BP-degrading microorganisms in different soils from cultivated fields. In this study, chemical properties, degradation behavior of the PBSA film, microbial populations, and esterase activities of 11 soil samples from different cultivated fields in Japan were analyzed under laboratory condition.

## Materials and methods

### Substrate and chemicals

To select the PBSA-degrading microorganisms from soils, emulsified PBSA (Bionolle® EM-301; average molecular weight, 12 to 15 × 10^4^; Showa Denko K. K., Tokyo, Japan) was used as substrate. To evaluate their solid polymer-degrading activity, black PBSA film (Bionolle® 3001 G, Showa Denko K. K.) was used. It contains carbon black as additive, and has an average molecular weight of 20 to 25 × 10^4^ and a thickness of 20 μm. *p-*Nitrophenol valerate (*p*NP-valerate) was purchased from Sigma-Aldrich (St. Louis, MO, USA) for the assay of esterase activity.

### Soil analysis

Soils collected from the plowed layers of 11 cultivated fields in Japan (Table 1) were stocked at 4°C before use. For chemical analysis, these soil samples were air-dried and sieved through a 2-mm mesh. Water content was determined by drying 10 g of soil sample in an oven at 105°C overnight. To measure soil pH(H_2_O), 2 g of soil sample were mixed with 5 ml of deionized water and allowed to stand for 1 d, after which the pH(H_2_O) of the suspension was measured using a standard pH meter (F-23II; Horiba, Ltd., Kyoto, Japan). For the determination of total carbon (C) and nitrogen (N) contents in the soils, visible plant residues in the soil samples were carefully removed by using tweezers. The soil samples were thoroughly ground using a mortar and subjected to NC analysis (Sumigraph NC-22 F, Sumika Chemical Analysis Service, Osaka, Japan).

**Table 1:** **Properties of soils used in this study**

**Soil sample**	**Soil type**	**Soil texture**	**pH(H**_**2**_ **O)**	**Total carbon (%)**	**Total nitrogen (%)**	**Area of soil sampling**
CHI	alluvial	sandy loam	7.17	1.30	0.13	Chiba
TKB	volcanic ash	loam	6.30	6.22	0.59	Ibaraki
HIO	alluvial	loam	7.05	2.08	0.17	Kagoshima
MJO	volcanic ash	sandy loam	5.36	6.95	0.48	Miyazaki
TAK	alluvial	sandy loam	7.30	NA*	NA	Miyazaki
KIB	alluvial	clay loam	6.15	3.37	0.25	Okayama
OKA	alluvial	loam	7.14	4.52	0.39	Okayama
AKA	alluvial	loam	5.78	1.39	0.10	Shimane
MIY	alluvial	sandy loam	5.91	0.97	0.09	Shimane
YM1	volcanic ash	clay loam	7.26	2.61	0.27	Yamanashi
YM2	alluvial	clay loam	7.17	2.07	0.22	Yamanashi

*NA: not analyzed.

### Degradation assay of PBSA films in soils

The degree of degradation of the PBSA film in the soil was evaluated using the procedure used in our previous study (Kitamoto et al. 2011), with modifications as follows. Fresh sieved soil samples were used for the analysis after being brought to a water content of 50% (w/w) of maximum water holding capacity. Pieces of the PBSA film (2 × 2 cm) were packed in between two layers of moistened soil (20-g lower layer and 20-g upper layer) in a sterilized plastic petri dish (φ90 × D20 mm) and incubated at 25°C. The dishes were wrapped with parafilm, and packaged in polyethylene bags in order to keep the moisture during the entire investigation periods. Three dishes with four pieces of film in each were prepared. For the control, sterilized soil was prepared by autoclaving (121°C, 15 min) or gamma ray irradiation (30 kGy).

One piece of film was collected from each dish at 1-week interval for 4 weeks. The mean degradation ratio of three pieces of film collected each time was calculated from mean gray values of digital images containing each collected piece. An image of the residual black film was scanned with a film scanner and saved in TIFF format (300 dpi). A mean gray value (from completely black = 0 to completely white = 255) of 300 × 300 pixels containing an image of residual film was compared with that of a fresh film by the Image J (Schneider et al. 2012). We then obtained threshold values from the mean gray value of the background image without the film from each image files. Degradation ratio (%) was calculated using the following equation:

$$ Degradation\  ratio\ \left(\%\right)=\frac{\left( gray\  value\  of\  residual\  film\right)-\left( gray\  value\  of\  fresh\  film\right)}{\left( gray\  value\  of\  background\right)-\left( gray\  value\  of\  fresh\  film\right)}\times 100 $$

To represent the degradation speed of a film in each soil, the degradation rate (ratio/week) was calculated as average degradation ratio during the first 3 weeks when our results showed linear increase in the degradation ratio.

### Media and cultural conditions and microbial viable counts

Soils adhering to the PBSA film during the degradation tests were collected with sterilized spoon and used as peripheral soil for various analyses. Viable counts of microorganisms in the field soil and peripheral soil of the PBSA film were carried out on solid agarose media, and determined as colony forming unit (CFU) of soil suspension. The soil suspension was prepared from one gram of wet soil sample by shaking it in 10 ml of distilled water at 25°C for 10 minutes at 160 rpm. The suspension was diluted with distilled water, and spread on two kinds of selective agarose media: RFMM (fungal minimal medium with rose bengal) and DNB (diluted nutrient broth), designed for determination of total viable counts of fungi and bacteria, respectively. The RFMM agar (liter^-1^) was composed of 2 g NaNO_3_, 0.2 g MgSO_4_·7H_2_O, 0.2 g KH_2_PO_4_, 1 g yeast extract, and 15 g agar dissolved in tap water before autoclaving. After autoclaving, 40 μg ml^−1^ chloramphenicol and 33 μg ml^−1^ rose bengal were added aseptically to the medium to inhibit growth of bacteria and fungi, respectively. The DNB agar was composed of commercial nutrient broth (Difco, Becton, Dickinson and Company, Franklin Lakes, New Jersey) diluted 100-fold with distilled water, and then added with 15 g liter^-1^ agar. It was added with cycloheximide (50 μg ml^−1^) to inhibit growth of eukaryotic microorganisms.

Microorganisms, which degrade emulsified PBSA, from soil suspensions were evaluated on two kinds of double layered-selective media (RFMM and DNB). To form the bottom layer, 15 ml of each medium with appropriate antibiotics were poured onto separate plates. Upon solidification, each plate was poured with 10 ml of solution (upper layer) containing 1% (w/v) emulsified PBSA, and 1.5% (w/v) agarose. The RFMM and DNB plates were incubated at 25°C for 4 days and 7 days, respectively. The microbial counts were calculated as CFU per gram of dry soil.

### Identification of microorganisms

The microorganisms that grew on plates were observed under the microscope to distinguish their morphological characteristics; and they were identified based on 5.8S rDNA-ITS (for fungi) sequences as described previously (Marchesi et al. 1998; White et al. 1990).

### Soil esterase assay

Esterase activity for *p*-nitrophenyl acetate (*p*NP-acetate) in the soil can be used as an indicator of poly(butylene succinate) biodegradation (Sakai et al. 2002). However, in our additional experiment, the esterase activity in the OKA and TKB soils with PBSA with *p*NP-acetate as substrate showed lower increase than those that with *p*NP-valerate (data not shown). Furthermore, the previously reported biodegradable polyester-degrading enzymes from bacteria (Akutsu-Shigeno et al. 2003) and fungi (Kodama et al. 2009; Maeda et al. 2005; Shinozaki et al. 2013; Suzuki et al. 2012) preferred to hydrolyze longer-chain esters of *p*NP rather than *p*NP-acetate. These observations suggest that esterases production by microorganisms present in the soils tested increased in the samples with PBSA, and that they preferred to degrade *p*NP-valerate, like the other microbial esterases identified previously. Considering these results, we therefore estimated soil esterase activities with *p*NP-valerate instead of *p*NP-acetate as substrate in the present study. Esterase activity was assayed as described in a previous study (Sakai et al. 2002) with modifications as follows: moist soil samples (100 mg) were incubated with ester substrate (2 mmol liter^-1^*p*NP-valerate) in 0.6 ml tris(hydroxymethyl) aminomethane (Tris)-maleic buffer (0.5 mol liter^-1^, pH 6.0) in 2-ml plastic tubes. The tubes were shaken continuously during incubation at 15 rpm with a shaker (RT-30mini, TAITEC Co. Ltd, Saitama, Japan) at 30°C for 30 min. After incubation, they were centrifuged at 20,000 × *g* for 5 min, and from each tube, 75 μl of supernatant was collected and was mixed with 200 μl of 100% ethanol. Then 55 μl of 2 mol liter^-1^ Tris was added to the mixture and vortexed for a few seconds. The absorbance of the mixture was measured at 405 nm by multi-spectrophotometer (Benchmark Plus, Bio-Rad Laboratories, Hercules, CA, USA). The absorbance of *p*NP-valerate in the buffer without soil and that of each soil extract mixture without the substrate were subtracted as a blank and as a background, respectively, from the measured absorbance of the mixture. The data shown represent geometric means of at least three independent assays.

### Statistical analysis

All statistical analyses were performed by R program (version 2.15.0) (R Development Core Team 2012). For statistical testing of normality, the Kolmogorov-Smirnov test was used. The Spearman rank correlations were calculated to assess the correlation between the isolation rate of PBSA-degrading fungi and degradation speed of PBSA film in the soil; the difference with *P* < 0.05 was considered significant. Data for viable counts of the microorganisms were normalized by logarithmic transformation, and nontransformed values were presented in the Results. The changes in the population of the PBSA-degrading fungi present in the peripheral soil of the PBSA film were examined by Williams’ test (Williams 1971, 1972) for multiple comparisons with a source code (Aoki 2004). The increase in the esterase activities during the degradation of the PBSA film in soils were analyzed by the Shirley-Williams’ test (Shirley 1977) for multiple comparisons with a source code (Aoki 2012). Williams’ test and the Shirley-Williams’ test were conducted at the one-tailed significance level of 2.5% (α = 0.025). Statistical difference in the esterase activity between the soils with and without the PBSA film at 3 weeks after incubation was determined by two-sided Mann-Whitney’s *U*-test.

## Results

### Soil characterization

Soil characteristics, such as type, texture, soil pH(H_2_O), total C and N contents, as well as the sampling area of the tested soils are presented in Table 1. All soil samples were found to be either alluvial soil or volcanic ash soil, with textures ranging from sandy loam to clay loam. The soil pH(H_2_O) values ranged from 5.78 to 7.26. Soil sample TKB had the highest total C and N contents, and sample MIY, the lowest.

### Visual observation of the PBSA film degradation in different soils

**Figure 1:**
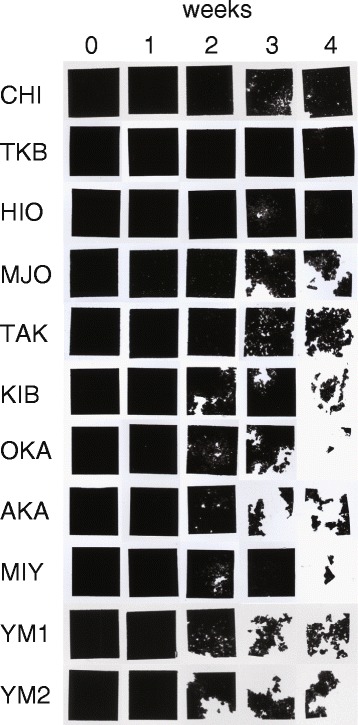
**Degradation of the PBSA film buried for 4 weeks in soils from 11 cultivated fields.**

Degradation speed of soil-buried PBSA films varied with the soil samples. However, by at least 2 weeks of incubation, tiny holes, tears, or thinned areas were observed in all the incubated films (Figure 1). The weekly degradation ratio of film in each soil sample, covering a 4-week incubation period is presented in Table 2. Among all tested soils, the soil sample OKA showed the highest degradation ratio of films as well as the highest degradation speed after 4 weeks of incubation. The degradation speed of the PBSA film incubated in soil sample TKB was the slowest of all. Films incubated in autoclaved or gamma ray-irradiated soils were not usually degraded, but after a few weeks, some of them showed signs of degradation which could be attributed to incomplete sterilization of soil (data not shown).

**Table 2:** **Degradation ratios (%) of soil-buried PBSA films**

**Soil sample**	Degradation ratio (%)*			
	**Incubation period (week)**			
	**1**	**2**	**3**	**4**
CHI	−2.4 ± 2.5	−0.9 ± 0.9	7.4 ± 7.3	33.1 ± 9.0
TKB	1.0 ± 3.5	−0.7 ± 1.3	0.8 ± 1.5	1.4 ± 3.4
HIO	−1.7 ± 1.1	1.2 ± 1.4	−0.2 ± 0.2	7.3 ± 8.1
MJO	0.6 ± 1.7	0.6 ± 3.0	4.0 ± 2.0	7.9 ± 1.8
TAK	1.8 ± 2.5	3.3 ± 1.7	5.4 ± 3.2	30.1 ± 6.6
KIB	−4.0 ± 2.1	7.8 ± 5.7	34.5 ± 8.3	58.9 ± 36.3
OKA	0.4 ± 2.6	3.9 ± 5.8	60.3 ± 23.2	95.9 ± 6.6
AKA	−4.7 ± 2.5	0.5 ± 2.3	54.3 ± 22.9	66.0 ± 17.3
MIY	1.2 ± 0.2	5.2 ± 3.2	28.5 ± 27.7	69.7 ± 33.2
YM1	2.7 ± 0.9	16.9 ± 3.4	62.0 ± 14.1	48.5 ± 11.6
YM2	0.9 ± 1.0	21.4 ± 5.0	44.1 ± 9.2	61.3 ± 8.4

*The ratios represent the means ± standard error of triplicate assays.

Degradation ratios were calculated by image scanning method as indicated in the equation given in the Materials and methods section.

### Microbial viability in different soil samples

Viable counts of the total culturable microorganisms and the PBSA degraders in each soil sample are listed in Table 3. Based on the results on RFMM agarose plate culture, the fungal population in 11 soil samples ranged from 2.88 × 10^4^ to 2.10 × 10^5^ CFU g^−1^, while the population of PBSA emulsion-degrading fungi ranged from 2.74 × 10^3^ to 8.12 × 10^4^ CFU g^−1^. These results demonstrate that PBSA-degraders constituted 4.1 to 42.3% of the total fungal populations. In many cases, lower counts of PBSA degraders were isolated from DNB plate containing cycloheximide compared to those from RFMM with chloramphenicol. We expected to isolate PBSA-degrading bacteria on DNB plates containing cycloheximide. However, most of the microorganisms that formed clear zone around their colonies on the plates were confirmed under the microscope that they were not bacteria, and their 5.8S rDNA-ITS sequences [DDBJ: LC009007, and LC009008] showed the highest similarity (99%) to *Purpureocillium lilacinum* (formerly *Paecilomyces lilacinus*). This species has been reported as a cycloheximide-resistant fungus (Ali-Shtayeh et al. 1998). Under the conditions in this study, it was found that fungi were predominantly responsible for emulsified PBSA degradation. *Purpureocillium* strains were isolated from different soil samples; TKB, HIO, MJO, and OKA.

**Table 3:** **Viable counts of the total microorganisms and the PBSA degraders, and the isolation rates**

**Soil**	**Medium for screening fungi**			**Medium for screening bacteria**		
	**Total (CFU g** ^**−1**^ **)**	**PBSA degrading (CFU g** ^**−1**^ **)**	**Colonies clearing PBSA (%)**	**Total (CFU g** ^**−1**^ **)**	**PBSA degrading (CFU g** ^**−1**^ **)**	**Colonies clearing PBSA (%)**
CHI	6.28 × 10^4^	1.55 × 10^4^	24.7	4.37 × 10^7^	2.83 × 10^3^	0.0
TKB	6.06 × 10^4^	8.47 × 10^3^	14.0	2.59 × 10^7^	2.49 × 10^4^	0.1
HIO	6.41 × 10^4^	2.63 × 10^3^	4.1	NA*	NA	NA
MJO	2.10 × 10^5^	8.12 × 10^4^	38.6	3.86 × 10^7^	0.0	0.0
TAK	9.48 × 10^4^	1.45 × 10^4^	15.3	1.82 × 10^8^	6.13 × 10^2^	0.0
KIB	1.01 × 10^5^	2.71 × 10^4^	26.8	NA	NA	NA
OKA	4.08 × 10^4^	1.72 × 10^4^	42.3	5.78 × 10^7^	9.66 × 10^3^	0.0
AKA	5.88 × 10^4^	8.99 × 10^3^	15.3	NA	NA	NA
MIY	2.88 × 10^4^	2.74 × 10^3^	9.5	NA	NA	NA
YM1	6.09 × 10^4^	2.45 × 10^4^	40.3	2.82 × 10^7^	2.95 × 10^2^	0.0
YM2	5.62 × 10^4^	2.88 × 10^3^	51.3	2.53 × 10^7^	4.64 × 10^3^	0.0

*NA: not analyzed.

### Correlation between the isolation rate of PBSA-degrading fungi and degradation speed of PBSA film in the soil

**Figure 2:**
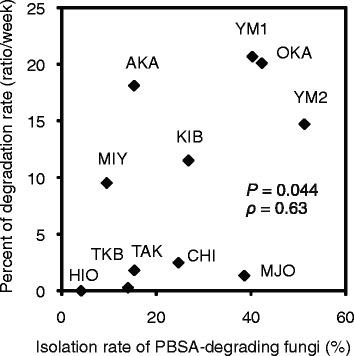
**The isolation rate of the PBSA-degrading fungi and the degradation rate of the PBSA film.** The scatter diagram shows the correlation between the isolation rate of the PBSA-degrading fungi and the degradation rate of the PBSA film in different soils. *P* represents the significance, and ρ represents Spearman's correlation coefficient. Degradation rate (%) is the average degradation during the first 3 weeks (ratio/week).

The isolation rates of the PBSA-degrading fungi were shown to be correlated with the degradation speed of the PBSA film (Figure 2). Soil sample with higher isolation rate for PBSA-degrading fungi showed a tendency toward higher degradation speed (Spearman’s *ρ* = 0.63, *P* = 0.044).

### Correlation between the isolation rate of PBSA-degrading fungi and esterase activity in the soil

**Figure 3:**
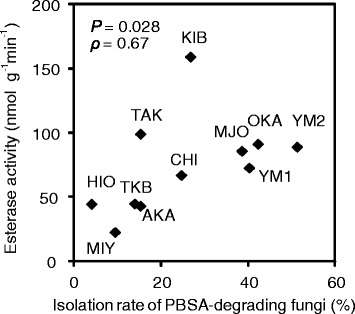
**The isolation rate of the PBSA-degrading fungi and the esterase activity.** The scatter diagram shows the correlation between the isolation rate of the PBSA-degrading fungi and the esterase activity in different soils. *P* represents the significance, and ρ represents Spearman's correlation coefficient.

The isolation rates of the PBSA-degrading fungi were also found to be correlated with the esterase activities in the different soils (Spearman’s *ρ* = 0.67, *P* = 0.028) (Figure 3). The highest esterase activity was recorded in soil sample KIB (159.3 nmol g^−1^ min^−1^), and the lowest was found in MIY (22.9 nmol g^−1^ min^−1^).

### Effect of soil-buried film on the population of the PBSA-degrading fungi and esterase activity in peripheral soils

**Figure 4:**
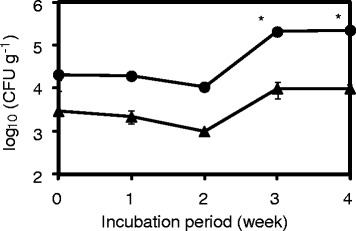
**Changes in the viable counts of the PBSA-degrading fungi in peripheral soils for 4 weeks.** The PBSA film buried in OKA (circles) and TKB (triangles) soil samples. The data shown are geometric means with error bar (1 standard deviation) of triplicate assays. Asterisk represents a significant increase (α = 0.025) in the population of the PBSA-degrading fungi from the 0-day control of each soil sample.

Viable counts of the PBSA-degrading fungi in the peripheral soils of the PBSA film buried in two soil samples (OKA and TKB) for 4 weeks are presented in Figure 4. Significant increase in PBSA degrader populations was detected after 3 and 4 weeks of incubation in OKA. The population of the PBSA-degrading fungi isolated from OKA was higher than that from TKB in all the sampling periods.

**Figure 5:**
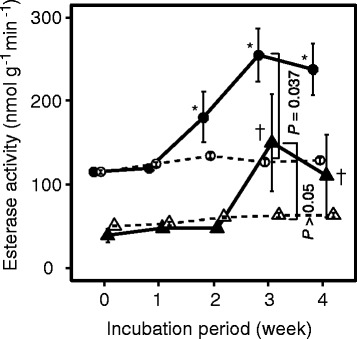
**Changes in the esterase activities in soil for 4 weeks with or without PBSA.** The peripheral soils of the PBSA film: OKA (black circles) and TKB (black triangles); the no treatment soils (without PBSA film) for control: OKA (white circles) and TKB (white triangles). The data represent geometric means with error bar indicating 1 standard error of 6 independent assays. Asterisk and dagger represent a significant increase (α = 0.025) in the esterase activity from the 0-day controls of each soil sample. *P* values represent significance levels between the esterase activity in the soils with and without PBSA film.

The esterase activities of these two soils monitored at each sampling period are shown in Figure 5. A significant increase of the esterase activity was detected in both soil samples with PBSA during the incubation periods. The basal esterase activity was higher in OKA than in TKB. In OKA, significant differences in esterase activities were detected between treatments with and without PBSA at 3 weeks of incubation (Mann-Whitney’s *U*-test, *P* = 0.037). Without buried PBSA film, the esterase activity of each soil sample was found to be stably low.

## Discussion

In agricultural fields in Japan, the degradation speed of BP mulch films is sometimes substantially early or slower than what is desired for practical use, and that hold back farmers from using BP mulch films in place of non-degradable ones. In this study, we showed the correlations between BP film degradation rate in cultivated soil and esterase activity and ratio of the PBSA-degrading soil fungi in the total soil fungal population. No PBSA-degrading bacteria have been isolated from the same soil samples. We were not able to find any significant correlation between the analyzed soil characteristics (pH[H_2_O], total carbon and nitrogen content) and the degradation rate of the PBSA films, esterase activities, and the isolation rates of the PBSA-degrading fungi. Aliphatic polyesters can be degraded non-enzymatically via simple chemical hydrolysis in the environment (Vert 2005). However, the lower degradation speed of the PBSA film in sterilized soil compared to that in unsterilized one after four weeks incubation also support our contention that PBSA film degradation is mainly caused by the polyester-degrading activity of enzymes produced by soil microorganisms rather than by non-enzymatic chemical hydrolysis. Further studies are expected to provide information about the chemical and physical characteristics of soils that influence BP film degradation speed. The possibility remains that there are some undetected characteristics of soils that promote BP degradation by soil microorganisms.

We have previously reported that 2 ~ 100% of yeast populations isolated from rice husks (Kitamoto et al. 2011) and 4.5% of fungal strains isolated from gramineous plants (Koitabashi et al. 2012) degrade PBSA emulsion. Similarly, previous investigators reported that fungi are the major degraders of BPs, including poly(3-hydroxybutyrate-*co*-3-hydroxyvalerate) (Sang et al. 2002), polyester polyurethane (Barratt et al. 2003; Cosgrove et al. 2007), and poly(butylene adipate*-co-*terephthalate) (Kasuya et al. 2009) in soil environments. Nowak *et al.* (2011) reported a higher increment and diversity of fungal population in the soil containing PBSA film compared to that of soil bacteria. Their results also support our observation that soil fungi greatly contribute to BP film degradation.

We found a significant correlation between the isolation rate of the PBSA-degrading fungi and the degradation rate of the PBSA film in the field soil samples (Figure 2). Likewise, the isolation rate of the PBSA-degrading fungi was shown to be significantly correlated with the esterase activity (Figure 3). These results indicate that the degradation of biodegradable mulch films in the soils is strongly influenced by its native distribution ratio of the PBSA-degrading fungi in the soils. The soil sample containing higher population ratio of PBSA-degrading fungi (OKA) showed relatively higher basal esterase activities (Figure 5). These extracellular esterases are expected to break the ester bonds of plant residues and other natural materials in the soils, as well as of BPs, thus, providing the necessary nutrients for the growth of soil microorganisms during cultivation.

Degradation ratios of soil-buried PBSA films were found to be not highly correlated with soil esterase activity. In this study, we measured soil esterase activity by using *p*NP-valerate as substrate. Some soil microorganisms produce a variety of enzymes having esterase activity with specific substrate preferences. For example, a cutinase of *Fusarium solani* prefers *p*NP-butyrate to *p*NP-acetate as substrate, and cutinase-like enzyme of *Cryptococcus* sp. S-2 prefers *p*NP-caproate to *p*NP-butyrate and *p*NP-acetate (Kodama et al. 2009). The enzymes substrate spectrum, optimum temperature, pH and other conditions are expected to be varied as well. The abundance ratio of PBSA-degrading esterases in each soil is still unknown. Currently, we are trying to evaluate PBSA-degradation activities in different soils.

Burying PBSA films in the soils stimulated the esterase production through enhanced proliferation of the PBSA degraders during the incubation period (Figures 4 and 5). Quicker and more drastic increase of esterase activity in the OKA soil sample compared to that in TKB is attributed to the larger distribution ratio of the basal PBSA degraders in the former than in the latter, resulting in the increase in the total esterase activity in the OKA soil.

This study has confirmed our knowledge that fungi contribute to mulch film-degradation in cultivated soils under laboratory conditions. A high isolation rate of PBSA-degrading fungi in cultivated soil could potentially serve as an indicator of the soil’s ability to promote BP film degradation. In the light of our findings, there is a need to conduct further studies in order to identify other physical and chemical properties of soil that greatly affect the speed of enzymatic degradation of BP film in soil environments.

## Abbreviations

BP: Biodegradable plastic
C: Carbon
CFU: Colony forming unit
DNB: Diluted nutrient broth
N: Nitrogen
PBSA: Poly(butylene succinate-co-adipate)
*p*NP: *p*-nitrophenol
*p*NP-acetate: *p*-nitrophenyl acetate
*p*NP-valerate: *p-*nitrophenyl valerate
rDNA: ribosomal deoxyribonucleic acid
rDNA-ITS: rDNA internal transcribed spacers
RFMM: Fungal minimal medium with rose bengal
Tris: Tris(hydroxymethyl) aminomethane

## References

[CR1] Akutsu-Shigeno Y, Teeraphatpornchai T, Teamtisong K, Nomura N, Uchiyama H, Nakahara T, Nakajima-Kambe T (2003). Cloning and sequencing of a poly(DL-lactic acid) depolymerase gene from *Paenibacillus amylolyticus* strain TB-13 and its functional expression in *Escherichia coli*. Appl Environ Microbiol.

[CR2] Ali-Shtayeh MS, Jamous RM, Abu-Ghdeib SI (1998). Ecology of cycloheximide-resistant fungi in field soils receiving raw city wastewater or normal irrigation water. Mycopathologia.

[CR3] Aoki S (2004) Williams no houhou ni yoru taju hikaku. (Williams’ multiple comparison test). http://aoki2.si.gunma-u.ac.jp/R/Williams.html Accessed 13 January 2015

[CR4] Aoki S (2012) Shirley-Williams no houhou ni yoru taju hikaku. (Shirley-Williams’ multiple comparison test). http://aoki2.si.gunma-u.ac.jp/R/Shirley-Williams.html Accessed 13 January 2015

[CR5] Barratt SR, Ennos AR, Greenhalgh M, Robson GD, Handley PS (2003). Fungi are the predominant micro-organisms responsible for degradation of soil-buried polyester polyurethane over a range of soil water holding capacities. J Appl Microbiol.

[CR6] Cosgrove L, McGeechan PL, Robson GD, Handley PS (2007). Fungal communities associated with degradation of polyester polyurethane in soil. Appl Environ Microbiol.

[CR7] Hoshino A, Sawada H, Yokota M, Tsuji M, Fukuda K, Kimura M (2001). Influence of weather conditions and soil properties on degradation of biodegradable plastics in soil. Soil Sci Plant Nutr.

[CR8] Kariyazono H, Nishimoto K, Hamaishi K (2000). Study of decomposition behavior of biodegradable plastic films in Kagoshima area soil. Rep Kagoshima Pref Inst Ind Technol.

[CR9] Kasuya K, Ishii N, Inoue Y, Yazawa K, Tagaya T, Yotsumoto T, Kazahaya J, Nagai D (2009). Characterization of a mesophilic aliphatic-aromatic copolyester-degrading fungus. Polym Degrad Stab.

[CR10] Kitamoto H, Shinozaki Y, X-h C, Morita T, Konishi M, Tago K, Kajiwara H, Koitabashi M, Yoshida S, Watanabe T, Sameshima-Yamashita Y, Nakajima-Kambe T, Tsushima S (2011). Phyllosphere yeasts rapidly break down biodegradable plastics. AMB Express.

[CR11] Kodama Y, Masaki K, Kondo H, Suzuki M, Tsuda S, Nagura T, Shimba N, Suzuki E, Iefuji H (2009). Crystal structure and enhanced activity of a cutinase-like enzyme from *Cryptococcus* sp. strain S-2. Proteins.

[CR12] Koitabashi M, Noguchi MT, Sameshima-Yamashita Y, Hiradate S, Suzuki K, Yoshida S, Watanabe T, Shinozaki Y, Tsushima S, Kitamoto HK (2012). Degradation of biodegradable plastic mulch films in soil environment by phylloplane fungi isolated from gramineous plants. AMB Express.

[CR13] Kyrikou I, Briassoulis D (2007). Biodegradation of agricultural plastic films: a critical review. J Polym Environ.

[CR14] Maeda H, Yamagata Y, Abe K, Hasegawa F, Machida M, Ishioka R, Gomi K, Nakajima T (2005). Purification and characterization of a biodegradable plastic-degrading enzyme from *Aspergillus oryzae*. Appl Microbiol Biotechnol.

[CR15] Marchesi JR, Sato T, Weightman AJ, Martin TA, Fry JC, Hiom SJ, Wade WG (1998). Design and evaluation of useful bacterium-specific PCR primers that amplify genes coding for bacterial 16S rRNA. Appl Environ Microbiol.

[CR16] Ngouajio M, Auras R, Fernandez RT, Rubino M, Counts JW, Kijchavengkul T (2008). Field performance of aliphatic-aromatic copolyester biodegradable mulch films in a fresh market tomato production system. Horttechnology.

[CR17] Nowak B, Pajak J, Drozd-Bratkowicz M, Rymarz G (2011). Microorganisms participating in the biodegradation of modified polyethylene films in different soils under laboratory conditions. Int Biodeterior Biodegrad.

[CR18] R Development Core Team (2012) R: a language and environment for statistical computing. R Foundation for Statistical Computing, Vienna, Austria. http://www.R-project.org/ Accessed 13 January 2015

[CR19] Sakai Y, Isokawa M, Masuda T, Yoshioka H, Hayatsu M, Hayano K (2002). Usefulness of soil *p-*nitrophenyl acetate esterase activity as a tool to monitor biodegradation of polybutylene succinate (PBS) in cultivated soil. Polym J.

[CR20] Sang BI, Hori K, Tanji Y, Unno H (2002). Fungal contribution to in situ biodegradation of poly(3-hydroxybutyrate-co-3-hydroxyvalerate) film in soil. Appl Microbiol Biotechnol.

[CR21] Schneider CA, Rasband WS, Eliceiri KW (2012). NIH Image to ImageJ: 25 years of image analysis. Nat Methods.

[CR22] Shinozaki Y, Morita T, Cao XH, Yoshida S, Koitabashi M, Watanabe T, Suzuki K, Sameshima-Yamashita Y, Nakajima-Kambe T, Fujii T, Kitamoto HK (2013). Biodegradable plastic-degrading enzyme from *Pseudozyma antarctica*: cloning, sequencing, and characterization. Appl Microbiol Biotechnol.

[CR23] Shirley E (1977). A non-parametric equivalent of Williams' test for contrasting increasing dose levels of a treatment. Biometrics.

[CR24] Suzuki K, Sakamoto H, Shinozaki Y, Tabata J, Watanabe T, Mochizuki A, Koitabashi M, Fujii T, Tsushima S, Kitamoto H (2012). Affinity purification and characterization of a biodegradable plastic-degrading enzyme from a yeast isolated from the larval midgut of a stag beetle, *Aegus laevicollis*. Appl Microbiol Biotechnol.

[CR25] Vert M (2005). Aliphatic polyesters: great degradable polymers that cannot do everything. Biomacromolecules.

[CR26] White TJ, Bruns ST, Lee SF, Taylor J, Innis MA, Gelfand DH, Sninsky JJ, White TJ (1990). Amplification and direct sequencing of fungal ribosomal RNA genes for phylogenetics. PCR protocols : a guide to methods and applications.

[CR27] Williams DA (1971). A test for differences between treatment means when several dose levels are compared with a zero dose control. Biometrics.

[CR28] Williams DA (1972). Comparison of several dose levels with a zero dose control. Biometrics.

